# Heat stress acutely activates insulin-independent glucose transport and 5′-AMP-activated protein kinase prior to an increase in HSP72 protein in rat skeletal muscle

**DOI:** 10.14814/phy2.12601

**Published:** 2015-11-05

**Authors:** Ayumi Goto, Tatsuro Egawa, Ichika Sakon, Rieko Oshima, Kanata Ito, Yasuhiro Serizawa, Keiichi Sekine, Satoshi Tsuda, Katsumasa Goto, Tatsuya Hayashi

**Affiliations:** 1 Laboratory of Sports and Exercise Medicine, Graduate School of Human and Environmental Studies, Kyoto UniversityKyoto, Japan; 2 Department of Physiology, Graduate School of Health Sciences, Toyohashi SOZO UniversityToyohashi, Japan

**Keywords:** Epitrochlearis, glucose metabolism, GLUT4, hyperthermia

## Abstract

Heat stress (HS) stimulates heat shock protein (HSP) 72 mRNA expression, and the period after an increase in HSP72 protein is characterized by enhanced glucose metabolism in skeletal muscle. We have hypothesized that, prior to an increase in the level of HSP72 protein, HS activates glucose metabolism by acutely stimulating 5′-AMP-activated protein kinase (AMPK). Rat epitrochlearis muscle was isolated and incubated either with or without HS (42°C) for 10 and 30 min. HS for 30 min led to an increase in the level of Hspa1a and Hspa1b mRNA but did not change the amount of HSP72 protein. However, HS for both 10 and 30 min led to a significant increase in the rate of 3-*O*-methyl-d-glucose (3MG) transport, and the stimulatory effect of 3MG transport was completely blocked by cytochalasin B. HS-stimulated 3MG transport was also inhibited by dorsomorphin but not by wortmannin. HS led to a decrease in the concentration of ATP, phosphocreatine, and glycogen, to an increase in the level of phosphorylation of AMPK*α* Thr^172^, and to an increase in the activity of both AMPK*α*1 and AMPK*α*2. HS did not affect the phosphorylation status of insulin receptor signaling or Ca^2+^/calmodulin-dependent protein kinase II. These results suggest that HS acts as a rapid stimulator of insulin-independent glucose transport, at least in part by stimulating AMPK via decreased energy status. Although further research is warranted, heat treatment of skeletal muscle might be a promising method to promote glucose metabolism acutely.

## Introduction

Heat stress (HS) has recently been implicated in the regulation of whole-body glucose homeostasis, and indications are that the upregulation of heat shock protein (HSP) 72 in skeletal muscle plays an important role in the mechanism leading to metabolic enhancement. Chung et al. (2008) showed that elevations of HSP72 protein in mouse skeletal muscle, which were induced by HS, transgenic overexpression, or pharmacological intervention with hydroxylamine derivative BGP-15, protected against diet- and obesity-induced insulin resistance and glucose intolerance. Gupte et al. (2009) demonstrated that upregulation of HSP72 protein by HS in rat skeletal muscle was associated with improved glucose tolerance and insulin sensitivity. Henstridge et al. (2014) showed that overexpressing HSP72 protein in mouse skeletal muscle improved glucose tolerance and insulin sensitivity as well as insulin-dependent glucose clearance in skeletal muscle. On the other hand, Drew et al. (2014) demonstrated the impairment of insulin-dependent glucose uptake in skeletal muscle and whole-body glucose intolerance in HSP72 knockout mice. Kurucz et al. (2002) reported decreased levels of expression of HSP72 mRNA in skeletal muscle in people with Type 2 diabetes mellitus (T2DM). Thus, there is substantial evidence to indicate that the amount of HSP72 protein in skeletal muscle is an important determinant for the regulation of whole-body glucose metabolism.

Although HS is a potent stimulator of HSP72 mRNA expression, it does not lead to a rapid increase in the amount of HSP72 protein in skeletal muscle. We have hypothesized that, prior to an increase in the level of HSP72 protein, HS activates glucose metabolism by rapidly stimulating 5′-AMP-activated protein kinase (AMPK), which is a metabolite-sensing kinase that is involved in mechanisms leading to insulin-independent glucose transport in skeletal muscle (reviewed in Friedrichsen et al. 2013; Richter and Hargreaves 2013). Our hypothesis is based on previous studies that have demonstrated the following: (1) HS seems to require more than 1 h to induce a significant increase in the amount of HSP protein in skeletal muscle (Oishi et al. 2002); (2) perfusion of dog hind-leg muscles with HS for 1 h induces a significant reduction in the energy status (i.e., a decrease in the concentration of ATP and phosphocreatine (PCr), and an increase in the concentration of AMP) (Ghussen and Isselhard 1984); (3) HS for 30 min in vivo and 10 min in vitro increases glucose transport in rat skeletal muscles (Koshinaka et al. 2013); (4) HS for 10 min in vitro increases AMPK*α* Thr^172^ phosphorylation in rat skeletal muscle (Koshinaka et al. 2013), and HS for 30–60 min increases AMPK*α* Thr^172^ phosphorylation in cultured muscle cells (Moon et al. 2003; Liu and Brooks 2012).

Skeletal muscle is the major site of whole-body glucose disposal, and the process of glucose transport across the cellular membrane is a rate-limiting step of glucose metabolism in skeletal muscle (reviewed in Huang and Czech 2007; Richter and Hargreaves 2013). To test our hypothesis, we investigated the short-term (<30 min) effect of HS on the levels of HSP72 mRNA and protein, and we examined the change in glucose transport activity together with underlying signaling mechanisms including AMPK. We used an isolated rat skeletal muscle preparation to eliminate potential confounders such as HS-induced alteration of muscle blood flow and concentration of humoral factors.

## Materials and Methods

### Animals

A total of 194 male Sprague–Dawley rats weighing 150–160 g (aged 5 weeks) was purchased from Shimizu Breeding Laboratories (Kyoto, Japan). Experimental protocols were approved by the Kyoto University Graduate School of Human and Environmental Studies, and by the Kyoto University Radioisotope Research Center, and followed the Guiding Principles for the Care and Use of Animals in the Field of Physiological Science (The Physiological Society of Japan, 2015).

### Muscle incubation

Muscle was treated as described previously (Toyoda et al. 2004) with modifications. Rats were fasted overnight before experiments. The rats were killed by cervical dislocation without anesthesia, and epitrochlearis muscles of each side were gently and rapidly (<1–2 min) dissected from nearly all the animals. Both ends of each muscle were immediately tied with sutures, and muscles were mounted on an incubation apparatus with the resting tension set to 0.5 g. To recover from *postmortem* AMPK activation induced during dissection (Toyoda et al. 2006), the muscles was preincubated in 7 mL of alpha minimum essential medium (*α*MEM) (Nacalai Tesque, Kyoto, Japan) supplemented with 0.01% bovine serum albumin (Sigma, St. Louis, MO), 2.2 g/L NaHCO_3_, 5 mmol/L mannitol, 2.54 mol/L CaCl_2_, 10% fetal bovine serum (Biowest, Nuaillé, France), 50 *μ*U/mL insulin, 0.005% Antifoam SI (Wako, Osaka, Japan), and 1% penicillin/streptomycin (Invitrogen, Carlsbad, CA) (Gupte et al. 2011) for 60 min maintained at 35°C. Muscles were then randomly assigned to the experimental groups. For HS treatment, muscles were then incubated in 7 mL of fresh medium maintained at 42°C for 10 or 30 min (Gupte et al. 2011). For maximal stimulation of AMPK, muscles were incubated in fresh medium containing 0.5 mmol/L 2,4-dinitrophenol (DNP) for 10 min or 2 mmol/L 5-aminoimidazole-4-carboxamide-1-*β*-d-ribonucleoside (AICAR) for 30 min at 35°C. For maximal stimulation of insulin signaling, muscles were incubated with 1 μmol/L insulin for 30 min at 35°C. When present, 25 μmol/L cytochalasin B (Nacalai Tesque), 4 μmol/L dorsomorphin (Nacalai Tesque), or 1 μmol/L wortmannin (Nacalai Tesque) was added in the preincubation and incubation buffer. The concentration of dimethyl sulfoxide was 0.5%, which had no effect in any assay.

For maximal stimulation of Ca^2+^/calmodulin-dependent protein kinase II (CaMKII) by tetanic contraction, muscles were preincubated in 7 mL of *α*MEM containing 1% penicillin/streptomycin for 40 min at 35°C. Muscles were then incubated in 7 mL of fresh medium for 30 min. Muscles were stimulated to contract during the last 10 min of incubation (train rate: 1/min, train duration: 10 sec, pulse rate: 100 Hz, pulse duration: 0.1 msec) by using an electric stimulator (SEN-3401; Nihon Kohden, Tokyo, Japan).

The muscles were used either fresh for glucose transport measurement or frozen in liquid nitrogen for subsequent analysis.

### Real-time reverse transcription

A separate set of muscle samples were subjected to Real-time reverse transcription (RT-PCR). Total RNA was extracted from frozen muscles by using the RNeasy Mini Kit (Qiagen, Venlo, the Netherlands). The RNA was reverse-transcribed into complementary DNA (cDNA) by using PrimeScript RT Master Mix (Perfect Real Time) (Takara Bio, Otsu, Japan).

HSP72 (Hspa1a and Hspa1b): Synthesized cDNA was subjected to real-time RT-PCR (Thermal Cycler Dice Real Time System II MRQ, Takara Bio) by using SYBR *Premix Ex Taq* II (Takara Bio), and then analyzed by using Takara Thermal Cycler Dice Real Time System Software Ver. 4.00. The PCR cycling conditions were at 95°C for 30 sec followed by 40 cycles at 95°C for 5 sec and at 60°C for 30 s. Amplification specificity was confirmed by melt-curve analysis. Rps18 cDNA was used as an internal standard for normalization of the amount of total RNA in each reaction. The primers were designed by using the Takara Bio Perfect Real Time Support System (Takara Bio). Primers used were as follows: Hspa1a, 5′-GGCGCTCCAGGTGTGATCTA-3′ (forward) and 5′-GACTTGATTGCAGACCGAACGA-3′ (reverse); Hspa1b, 5′-CCAGGCAGTAGGTCTGGTGATG-3′ (forward) and 5′-TGGATCCAAAGGCTTGAGGTG-3′ (reverse); Rps18, 5′-TTGGTGAGGTCAATGTCTGCTTT-3′ (forward) and 5′-AAGTTTCAGCACATCCTGCGAGT-3′ (reverse).

Glucose transporter (GLUT) 4: Synthesized cDNA was subjected to real-time RT-PCR (Step One Real Time System, Applied Biosystems, Carlsbad, CA) by using Power SYBR Green PCR Master Mix (Life technologies, Carlsbad, CA), and then analyzed by using StepOne Software v2.3. The PCR cycling conditions were at 95°C for 10 min followed by 40 cycles at 95°C for 15 sec and at 60°C for 60 sec. Amplification specificity was confirmed by melt-curve analysis. Rps18 cDNA was used as an internal standard for normalization of the amount of total RNA in each reaction. The primers were designed by using Invitrogen (Life Technologies). Primers used were as follows: GLUT4, 5′-CTCATGGGCCTAGCCAATG-3′ (forward) and 5′-GGGCGATTTCTCCCACATAC-3′ (reverse).

### Western blot analysis

A separate set of muscle samples were subjected to Western blot analysis, which was performed as described previously (Toyoda et al. 2004) with modifications. Frozen muscle was homogenized in ice-cold buffer (1:40 wt/vol) containing 20 mmol/L Tris·HCl (pH 7.4), 1% Triton X-100, 50 mmol/L NaCl, 250 mmol/L sucrose, 50 mmol/L NaF, 5 mmol/L sodium pyrophosphate, 2 mmol/L dithiothreitol, 4 mg/L leupeptin, 50 mg/L trypsin inhibitor, 0.1 mmol/L benzamidine, 1 mmol/L Na_3_VO_4_, and 0.5 mmol/L phenylmethylsulfonyl fluoride (buffer A) and centrifuged at 16,000 *g* for 40 min at 4°C. Denatured proteins were separated on polyacrylamide gel and transferred to polyvinylidene difluoride membranes. Membranes were blocked with nonfat dry milk and incubated with commercially available antibodies (HSP70/HSP72 [ADI-SPA-812; Enzo Life Sciences, Farmingdale, NY], Actin [#4968; Cell Signaling Technology, Danvers, MA], AMPK*α* Thr^172^ [#2531; Cell Signaling Technology], AMPK*α* [#2532; Cell Signaling Technology], acetyl-CoA carboxylase (ACC) Ser^79^ [07-303; Merck Millipore, Billerica, MA], ACC [#3662; Cell Signaling Technology], insulin receptor (IR) Tyr^1158/1162/1163^ [44-806G; Life Technologies, Carlsbad, CA], IR*β* [SC-711; Santa Cruz Biotechnology, Santa Cruz, CA], Akt Ser^473^ [#9271; Cell Signaling Technology], Akt [#9272; Cell Signaling Technology], GLUT4 [4670-1704, Biogenesis, Poole, UK], CaMKII Thr^286^ [#3361 or #12716; Cell Signaling Technology], CaMKII [#3362; Cell Signaling Technology]). Some membranes were incubated with a signal enhancer (Can Get Signal Immunoreaction Enhancer Solution, Toyobo, Tokyo, Japan). The membranes were then reacted with secondary antibody coupled to horseradish peroxidase, and developed with enhanced chemiluminescence reagents. The protein signals were detected with ImageCapture G3 (Liponics, Tokyo, Japan) or WSE-6100 LuminoGraph (ATTO, Tokyo, Japan).

### Glucose transport assay

A separate set of muscle samples were subjected to glucose transport assay, which was performed by using nonmetabolized 3-*O*-methyl-d-glucose (3MG), as described previously (Toyoda et al. 2004). After incubation, muscle was incubated in 2 mL of Krebs–Ringer buffer containing 1 mmol/L [^3^H]3MG (1.5 *μ*Ci/mL) (American Radiolabeled Chemicals, St. Louis, MO) and 7 mmol/L d-[1-^14^C]-mannitol (0.3 *μ*Ci/mL) (American Radiolabeled Chemicals) at 30°C for 10 min. Muscle was then digested with 1 mol/L NaOH at 80°C for 10 min, neutralized with 1 mol/L HCl, and centrifuged at 20,000 *g* for 3 min. Radioactivity in the supernatant was measured by dual-label liquid scintillation counting. The transport activity was expressed as micromoles of transported 3MG per milliliter of intracellular space per hour (Young et al. 1986).

### Isoform-specific AMPK activity assay

A separate set of muscle samples were subjected to isoform-specific AMPK activity assay. AMPK is comprised of a catalytic *α* subunit and two regulatory subunits, *β* and *γ*; two distinct *α* subunits (*α*1 and *α*2) are present in skeletal muscle. The kinase activities of *α*1-containing AMPK complex (AMPK*α*1) and *α*2-containing AMPK complex (AMPK*α*2) were measured as described previously (Toyoda et al. 2004). Frozen muscle was homogenized in buffer A, and supernatant (100 *μ*g of protein) was immunoprecipitated with the AMPK*α*1 or AMPK*α*2 antibody (Toyoda et al. 2004) and protein A-sepharose beads (Amersham Biosciences, Uppsala, Sweden). Kinase reactions were performed with SAMS peptide as substrate (Toyoda et al. 2004). Kinase activity was expressed as picomoles of incorporated ATP per minute per milligram of immunoprecipitated protein.

### ATP, PCr, and glycogen assay

A separate set of muscle samples were subjected to ATP, PCr, and glycogen assay. The ATP and PCr content was measured fluorometrically in perchloric acid extracts of muscle as described previously (Egawa et al. 2009). Glycogen content was assayed enzymatically as described previously (Nakano et al. 2006). ATP and PCr content is expressed as nanomoles per milligram wet weight of muscle, and glycogen content is expressed as nanomoles of glucose residues per milligram wet weight of muscle.

### Statistical analysis

**Figure 1:**
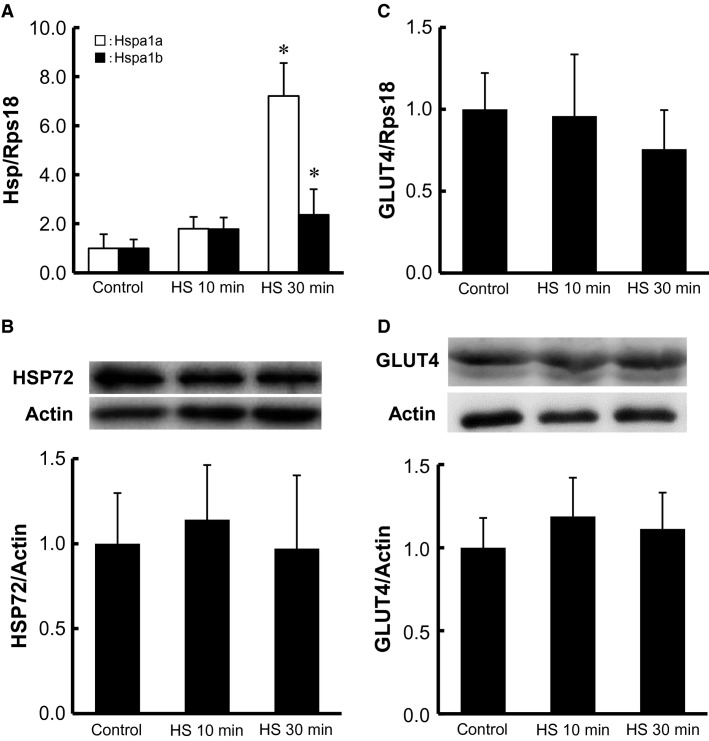
Short-term HS increases HSP mRNA but not HSP72 protein in isolated skeletal muscle. Isolated epitrochlearis muscle was incubated in medium for 0 (control), 10, or 30 min at 42°C. Muscle was assayed for Hspa1a and Hspa1b mRNA (A) or GLUT4 mRNA (C) with real-time RT-PCR, and HSP72 protein (B), or GLUT4 protein (D) with Western blot analysis. Fold increases are expressed relative to the level in muscles of the control group. The values are mean ± SD; *n *=* *5–6 (A), 9 (B), 5–6 (C), and 5–6 (D). *P < 0.05 versus corresponding control. Representative immunoblots are shown (B and D).

**Figure 2:**
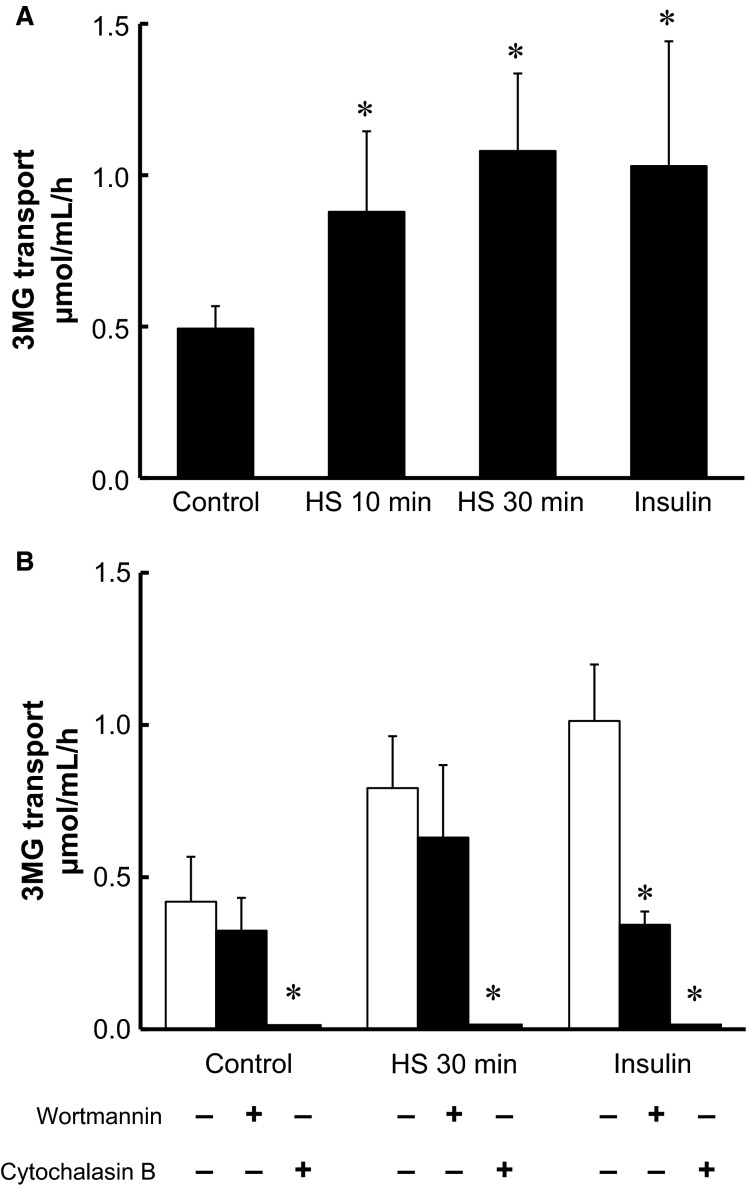
Short-term HS activates glucose transport in isolated skeletal muscle. Isolated epitrochlearis muscle was incubated in medium for 0 (control), 10, or 30 min at 42°C, or with 1 μmol/L insulin for 30 min at 35°C, then 3MG transport activity was determined (A). The values are mean ± SE; *n *=* *7–12. *P < 0.05 versus control. Isolated epitrochlearis muscle was incubated in medium for 0 (control) or 30 min at 42°C, or with 1 μmol/L insulin for 30 min at 35°C, in the absence or presence of either 1 μmol/L wortmannin or 25 μmol/L cytochalasin B, then 3MG transport activity was determined (B). The values are mean ± SD; *n *=* *5–7. *P < 0.05 versus corresponding basal.

**Figure 3:**
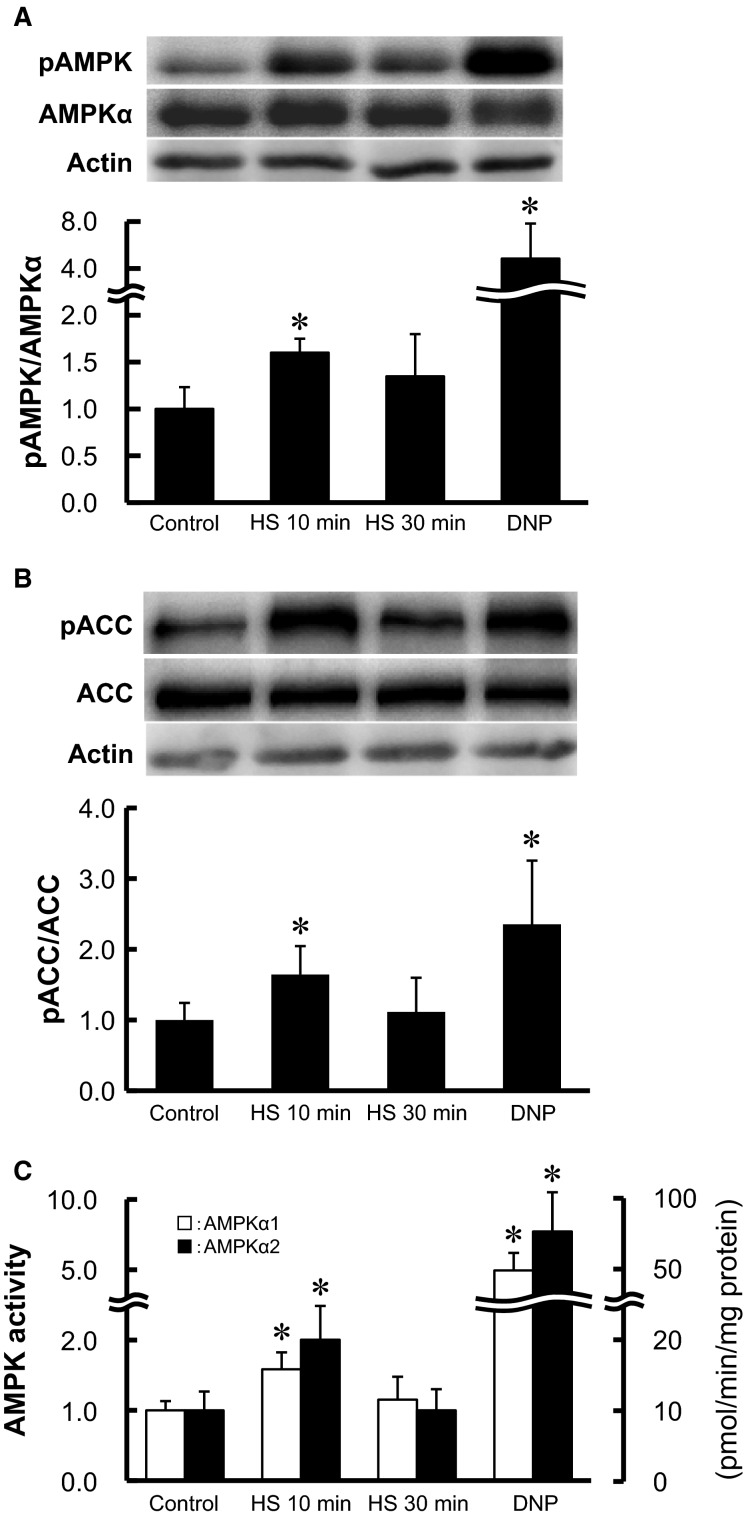
Short-term HS activates AMPK in isolated skeletal muscle. Isolated epitrochlearis muscle was incubated in medium for 0 (control), 10, or 30 min at 42°C, or with 0.5 mol/L DNP for 10 min at 35°C, then subjected to Western blot analysis for phosphorylated AMPK (A) or phosphorylated ACC (B), or isoform-specific AMPK activity assay (C). Fold increases are expressed relative to the level in muscles of the control group. The values are mean ± SD; *n *=* *4–13 (A), 6–14 (B), and 6–19 (C). *P < 0.05 versus corresponding control. Representative immunoblots are shown (A and B).

**Figure 4:**
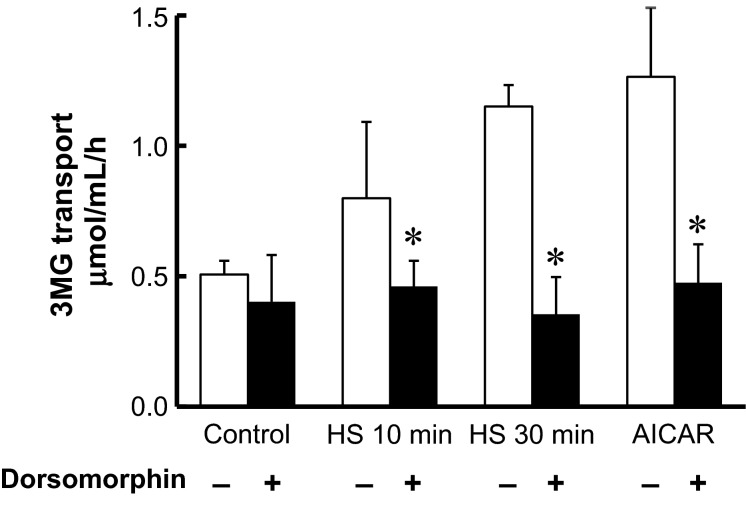
HS-stimulated glucose transport is inhibited by dorsomorphin in isolated skeletal muscle. Isolated epitrochlearis muscle was incubated in medium for 0 (control), 10, or 30 min at 42°C, or with 2 mmol/L AICAR for 30 min at 35°C, either in the absence or presence of 4 μmol/L dorsomorphin, then 3MG transport activity was determined. The values are mean ± SD; *n *=* *4–6. *P < 0.05 versus corresponding basal.

**Figure 5:**
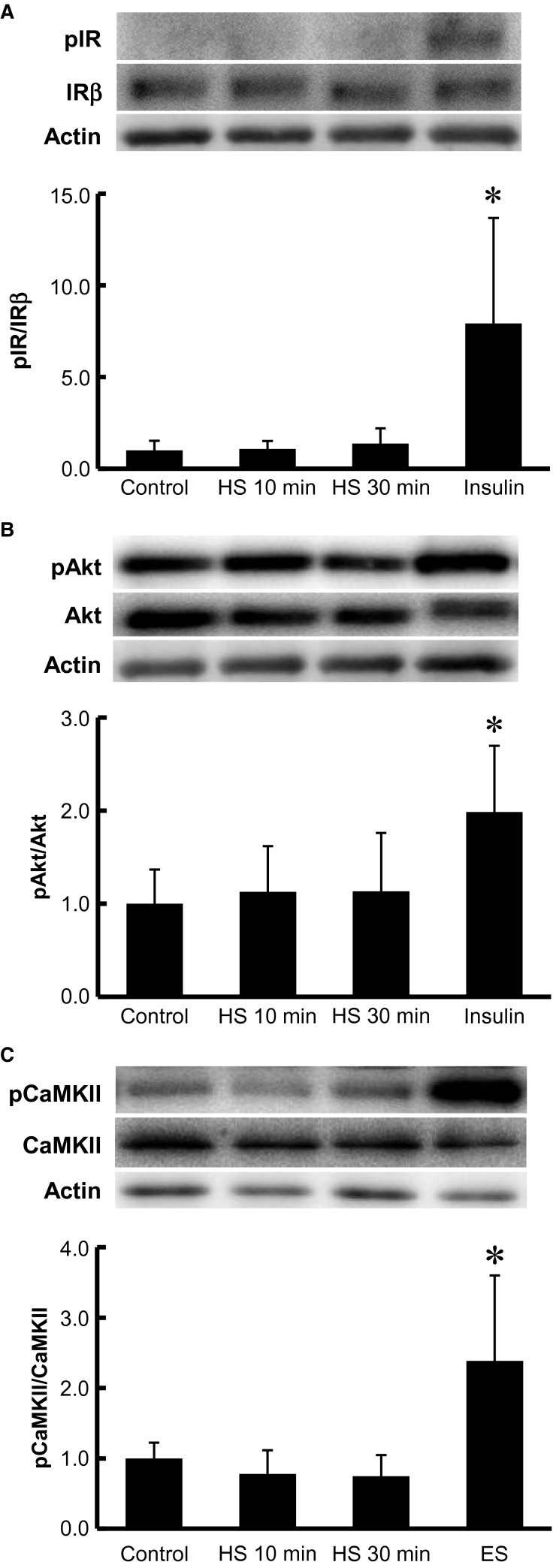
Short-term HS does not affect insulin signaling or CaMKII in isolated skeletal muscle. Isolated epitrochlearis muscle was incubated in medium for 0 (control), 10, or 30 min at 42°C. Muscle was also incubated in the presence of 1 μmol/L insulin for 30 min at 35°C or contracted by electrical stimulation (ES) at 35°C. Muscle was subjected to Western blot analysis for phosphorylated IR (A), phosphorylated Akt (B), or phosphorylated CaMKII (C). Fold increases are expressed relative to the level in muscles of the control group. The values are mean ± SD; *n *=* *5–6 (A), 6–7 (B), and 3–14 (C). *P < 0.05 versus corresponding control. Representative immunoblots are shown.

Data are expressed as mean ± standard deviation (SD). Differences between two groups (3MG transport with inhibitor vs. without inhibitor) were compared with Student’s *t*-test (Figs. 2B, 4). Multiple means were analyzed by using one-way ANOVA followed by post hoc comparison with Dunnett’s test (Figs.1, 2A, 3, 5 and Table1). *P* < 0.05 was considered statistically significant. All statistical analysis were performed using Ekuseru-Toukei 2012 software (Social Survey Research Information, Tokyo, Japan).

**Table 1:** Short-term HS decreases ATP, PCr, and glycogen content in isolated skeletal muscle

	Control	HS 10 min	HS 30 min
ATP	6.1 ± 0.7	3.9 ± 1.0*	2.7 ± 0.3*
PCr	14.5 ± 1.7	4.8 ± 1.3*	4.9 ± 1.6*
Glycogen	19.4 ± 1.2	11.8 ± 2.8*	10.4 ± 2.5*

Isolated epitrochlearis muscle was incubated in medium for 0 (control), 10, or 30 min at 42°C, and ATP, PCr, and glycogen content was determined. The values are mean ± SD; *n *=* *5–8.

* *P* < 0.05 versus control.

## Results

### Short-term HS increased expression of HSP mRNA, but not HSP protein

To confirm that acute HS stimulates HSP mRNA expression but does not increase the levels of HSP protein, we measured the amount of Hspa1a and Hspa1b mRNA by real-time RT-PCR analysis, and the amount of HSP72 protein by Western blot analysis. HSP72 is encoded for by the Hspa1a and Hspa1b genes, and we used an antibody that recognizes both HSPA1A and HSPA1B proteins in this study. We found that HS for 30 min led to a significant increase in the amount of both Hspa1a and Hspa1b mRNA by 7.2- and 2.4-fold, respectively (Fig.1A). In contrast, the same HS did not lead to a change in the amount of HSP72 protein (Fig.1B).

### Short-term HS increased glucose transport activity, which was inhibited by cytochalasin B, but not by wortmannin

HS for 10 and 30 min significantly increased 3MG transport by 1.8- and 2.2-fold, respectively, to a level comparable to that achieved by a maximally effective stimulation of insulin (Fig.2A). HS for 10 and 30 min did not change the amount of GLUT4 mRNA (Fig.1C) or GLUT4 protein (Fig.1D). Wortmannin, a phosphatidylinositol 3-kinase (PI3K) inhibitor, had no significant effect on basal 3MG transport, whereas it blocked insulin-stimulated glucose transport. In contrast, the inhibitor did not inhibit HS-stimulated glucose transport (Fig.2B). We also found that basal and HS-stimulated 3MG transport was totally inhibited by cytochalasin B, an inhibitory ligand of glucose transporters including GLUT4 (James et al. 1988) (Fig.2B). GLUT4 is the major isoform of glucose transporter in skeletal muscle, and it has been reported that the molecular ratio of GLUT4 to GLUT1 in the plasma membranes is 4:1 in the basal state and 7:1 in the insulin-stimulated state in rat skeletal muscle (Marette et al. 1992).

### Short-term HS increased both AMPK*α*1 and AMPK*α*2 activities and AMPK*α* Thr^172^ phosphorylation

Thr^172^ of the *α* subunits is the primary phosphorylation residue for AMPK activation. HS for 10 min increased phosphorylation of AMPK robustly (Fig.3A). HS for 10 min also increased the phosphorylation of ACC Ser^79^ (Fig.3B). ACC is an endogenous substrate of AMPK in skeletal muscle, and Ser^79^ phosphorylation of ACC reflects intracellular AMPK activity (Park et al. 2002). DNP, which is a known AMPK activator, also led to robust phosphorylation of both AMPK and ACC (Fig.3A and B). Similarly, the *α*-isoform-specific AMPK activity assay revealed that 10 min HS increased AMPK*α*1 and AMPK*α*2 activity by 1.6- and 2.0-fold, respectively (Fig.3C). However, the increases in AMPK phosphorylation, ACC phosphorylation, and *α*-isoform-specific AMPK activities were observed transiently and disappeared within 30 min after starting HS (Fig.3A, B, and C). The total AMPK*α* and ACC content did not change during the study (Fig.3A and B).

### Short-term HS-stimulated glucose transport was inhibited by dorsomorphin

Dorsomorphin (Compound C), an ATP-competitive inhibitor of AMPK (Zhou et al. 2001), had no significant effect on basal 3MG transport, whereas it completely blocked AICAR-stimulated glucose transport (Fig.4). Similarly, dorsomorphin blocked HS (for 10 and 30 min)-stimulated glucose transport completely. AICAR is transported into cells and phosphorylated to form ZMP, an AMP analog that has been shown to activate AMPK without changing the concentration of AMP (Hardie and Carling 1997).

### Short-term HS decreased ATP, phosphocreatine, and glycogen content

AMPK has been regarded as a “cellular fuel gauge” (Hardie and Carling 1997) and is activated by energy deprivation in skeletal muscle. To determine the effect of HS on the energy status, we measured the ATP, PCr, and glycogen levels in skeletal muscle. HS for 10 and 30 min decreased all these parameters significantly (Table1).

### Short-term HS did not increase the phosphorylation of insulin signaling molecules or CaMKII

We examined whether HS affects the activation status of insulin signaling in skeletal muscle. HS did not change the phosphorylation of IR (Fig.5A) or Akt (Fig.5B). CaMKII is activated by contraction and has been implicated in contraction-stimulated glucose transport in skeletal muscle (Wright et al. 2004; Witczak et al. 2010). We found that HS for 10 and 30 min (Fig.5C) as well as for 1 and 5 min (data not shown) did not affect CaMKII Thr^287^ phosphorylation.

## Discussion

Analysis of the data presented herein reveals novel findings in relation to the metabolic effects of HS in skeletal muscle. First, HS acutely increased insulin-independent 3MG transport prior to an increase in the level of HSP72 protein. Second, blockade of glucose transport by cytochalasin B indicates that glucose transport occurs through translocation of GLUT4. Third, HS reduced the muscle energy status and stimulated AMPK, and correspondingly, HS-stimulated 3MG transport was blocked by AMPK inhibitor. Lastly, HS did not activate insulin receptor signaling or CaMKII. Collectively, these results provide evidence that HS acts directly on skeletal muscle and acutely activates glucose metabolism, at least in part, through AMPK activation.

Energy deprivation is a strong stimulator of AMPK in skeletal muscle. In isolated rat skeletal muscle incubated in vitro, both AMPK*α*1 and AMPK*α*2 are activated by energy-decreasing stimuli, including exercise (contraction), hypoxia, chemical inhibition of oxidative phosphorylation, and hyperosmolarity (Hayashi et al. 2000), all of which lead to potent activation of insulin-independent glucose transport. Although the precise mechanism of reduced energy status by HS remains unclear, HS increased mitochondrial enzyme activity in rats (Chen et al. 1999) and oxygen consumption along with fatty acid oxidation in L6 muscle cells (Gupte et al. 2009). Thus, HS may act on energy status through an acceleration in the metabolic rate and an increase in the energy requirements rather than by inducing mitochondrial dysfunction and an inadequate energy supply.

We found that HS for 10 min enhances AMPK activity, which decreased rapidly within 30 min, whereas 3MG transport remained elevated (Figs.2, 3). However, previous reports have shown that AMPK activity is dissociated from glucose transport activity in skeletal muscle. Musi et al. (2001) demonstrated that tetanic contraction acutely stimulates AMPK activity and 3MG transport in isolated rat epitrochlearis muscle after electrical stimulation in vitro. They found that a single contraction for 10 sec is sufficient to begin to increase the activity of both AMPK*α*1 and AMPK*α*2 isoforms and 3MG transport, that maximal activity of AMPK and 3MG transport are found with 10 contractions, and that no further increase is observed with 15 contractions. They also found that the activity of both AMPK isoforms decreased rapidly after 10 contractions (*t*_1/2_ = 8 min), with a 70–80% decrease 10 min after contraction (Musi et al. 2001). In contrast, the rate of decrease in the level of 3MG transport was gradual, with no decrease being observed 10 min after contraction and only 50% decrease after 60 min. Correspondingly, studies of GLUT4 vesicle kinetics suggest that levels of plasma membrane GLUT4 is elevated immediately and 30 min after treadmill exercise in rat skeletal muscle, and that plasma membrane GLUT4 returns to baseline values within 2 h after exercise (Goodyear et al. 1990). In addition, our earlier report demonstrated that increased AMPK activity disappeared within 2 h after subcutaneous AICAR injection, while increased glucose transport activity persisted for at least 4 h in mouse muscles (Nakano et al. 2006). These observations suggest that, although AMPK plays a pivotal role in initiating increases in glucose transport in skeletal muscle, sustained enzyme activity is not necessary to preserve glucose transport activity. AMPK may be involved in triggering the translocation of GLUT4 into the plasma membrane by acute HS, but another mechanism, for example, reduction in GLUT4 endocytosis by novel PKCs (Li et al. 2014) might be responsible for maintaining transporters at the membrane.

We used an isolated muscle preparation to exclude potential influence by circulatory, humoral, and neural factors. We considered preceding studies that showed that muscle blood flow acts as an important determinant of the rate of glucose uptake in skeletal muscle (Baron et al. 1994) and is increased markedly by acute HS (Pearson et al. 2011). We also considered the preceding observation that HS to hind limbs in vivo for 30 min at 42°C increased plasma insulin concentrations 8.5-fold compared with control treatment at 36°C in rats (Koshinaka et al. 2013). In this study, the insulin concentration of the incubation medium was maintained at a physiological level (50 *μ*U/mL). Thus, the isolated muscle preparation made it possible to control the confounding components and to evaluate the direct effect of acute HS on skeletal muscle.

We demonstrated the effects of HS on epitrochlearis muscle but did not examine other muscles. Epitrochlearis is a fast-glycolytic muscle composed of approximately 65% fast-twitch white, approximately 20% fast-twitch red, and approximately 15% slow-twitch red fibers, and has been widely used for the study of glucose transport in skeletal muscle (Nesher et al. 1980; Wallberg-Henriksson and Holloszy 1985). Considering that the intracellular energy status is a critical regulator of AMPK activity (Hardie and Carling 1997), a range of muscle types, including slow-oxidative muscles, may respond to HS, although the magnitude of the response may vary. In fact, recent studies have demonstrated that acute energy deprivation caused by the application of various chemical compounds is associated with increased AMPK activity and glucose transport not only in epitrochlearis but also in soleus muscle in rat (Egawa et al. 2009; Ma et al. 2010; Tsuda et al. 2012; Serizawa et al. 2014; Oshima et al. 2015).

There are some reports that indicate the clinical relevance of HS for enhancing glucose metabolism in humans. For example, Hooper et al. (1999) treated eight human subjects with T2DM by immersing them in a hot water tub (37.8–41°C) up to their shoulders for 30 min, 6 days a week for 3 weeks. They found that fasting blood glucose and hemoglobin A1c levels decreased by 23 mg/dL and 1.0%, respectively. Biro et al. (2003) studied 25 subjects who had at least one of the conditions including hypertension, hyperlipidemia, T2DM, or obesity, and/or who smoked. Subjects were treated with a far-infrared sauna for 15 min and then with a warm blanket for 30 min, daily for 2 weeks. They showed a small but significant reduction in fasting blood glucose (99 to 94 mg/dL). Subsequent rodent studies also indicated the therapeutic usefulness of HS. Chung et al. (2008) found that a single exposure to HS (41.5°C, 15 min) is sufficient to increase the level of HSP72 protein in mouse skeletal muscle, and weekly treatment of HS (41.5°C, 15 min) for 16 weeks improved high-fat-diet-induced hyperinsulinemia and hyperglycemia in mice. Similarly, Gupte et al. (2009) showed that weekly HS treatment (between 41 and 41.5°C, 20 min) for 12 weeks improved glucose tolerance and insulin sensitivity in rats that were fed a high-fat diet, and this was associated with increased levels of HSP72 protein and enhanced insulin signaling in skeletal muscle. In contrast, our findings strongly suggest that a single exposure to HS can influence glucose metabolism acutely, prior to an increase in the amount of HSP72 protein. To our knowledge, no study has examined changes in muscle glucose metabolism during (or immediately after) exposure to HS in humans. We believe that our results provide a theoretical rationale for conducting future studies to investigate the acute effects of HS using various heat modalities in humans.

In summary, we have demonstrated for the first time that during the acute period after HS before an increase in the protein levels of HSP72, AMPK activity increases rapidly with decreased energy status, and insulin-independent glucose transport in skeletal muscle is activated. We propose that HS is an acute stimulus that promotes glucose metabolism, independently of increased HSP72 protein in skeletal muscle.
